# [Retracted] MicroRNA-27a functions as an oncogene in human osteosarcoma by targeting CCNG1 

**DOI:** 10.3892/ol.2026.15529

**Published:** 2026-03-16

**Authors:** Tao Lin, Quanping Ma, Yuefeng Zhang, Hongfei Zhang, Jiapeng Yan, Changhong Gao

Oncol Lett 15: 1067–1071, 2018; DOI: 10.3892/ol.2017.7389

Following the publication of this paper, it was drawn to the Editor's attention by a concerned reader that, regarding the cell invasion and migration assay data shown in Fig. 2A, the ‘inhibitor-NC / Migration’ data panel in the top row contained an overlapping section of data with the ‘miR-27a mimic / Invasion’ data panel in the bottom row, such that data which were intended to show the results of differently performed experiments had apparently been derived from the same original source. Moreover, upon performing an independent analysis of the data in this paper in the Editorial Office, it came to light that data featured in the ‘miR-27a mimic / Invasion’ data panel in the top row in Fig. 2 had already been submitted for publication in another paper written by different authors, also to the journal *Oncology Letters*.

In view of the fact that the abovementioned data had already apparently been submitted for publication prior to the receipt of this paper at *Oncology Letters*, the Editor has decided that this paper should be retracted from the Journal. The authors were asked for an explanation to account for these concerns, but the Editorial Office did not receive a reply. The Editor apologizes to the readership for any inconvenience caused.

